# Population structure, genetic diversity and core set construction of an international collection of 256 *Melissa officinalis* genotypes

**DOI:** 10.1186/s12870-026-08853-8

**Published:** 2026-05-01

**Authors:** Daniel von Maydell, Johannes Schwerdt, Heike Lehnert, Yvonne Pöschl-Grau, Thomas Schmutzer, Frank Marthe

**Affiliations:** 1 Institute for Breeding Research On Horticultural Crops, Julius Kühn Institute, Quedlinburg, Germany; 2 Institute of Agricultural and Nutritional Sciences, Chair of Plant Breeding, Martin Luther University Halle-Wittenberg, Halle, Germany; 3 Institute for Biosafety in Plant Biotechnology, Julius Kühn Institute, Quedlinburg, Germany

**Keywords:** Genetic variability, Germplasm, Lemon balm, Medicinal plant, Molecular markers

## Abstract

**Background:**

*Melissa officinalis* (balm) is a perennial medicinal. Climate change and high cultivation costs necessitate the breeding of new cultivars with improved stress tolerance and high metabolite content. However, the high costs of phenotyping large collections limit breeding progress. This study characterized the genetic diversity and population structure of 256 genotypes from 215 international accessions using flow cytometry and genotyping by sequencing (GBS). The primary objective was to identify untapped genetic resources and establish core sets to streamline future breeding and research efforts.

**Results:**

Morphological characterization and flow cytometry revealed a clear division by ploidy and subspecies. The collection comprised 209 diploid and three polyploid genotypes of ssp. *officinalis* (2C = 1.79 pg) and 44 tetraploid genotypes of ssp. *altissima* (4C = 3.57 pg). GBS generated 29,307 and 9,909 SNPs for the entire collection and a subset of ssp. *officinalis* genotypes, respectively. We identified significant genetic differentiation between the subspecies, as confirmed by PCA (PC1 = 69.9%), STRUCTURE, and hierarchical clustering. With 21,770 private alleles and H_E_ = 0.38 subspecies *altissima* exhibited greater genetic divergence than ssp. *officinalis* (2,953 private alleles, H_E_ = 0.07). Within ssp. *officinalis*, several clusters of untapped genetic diversity from Armenia, France, Georgia, and Spain were identified. Furthermore, genomic analysis revealed inconsistencies in varietal purity in three out of four investigated traditional cultivars, where genotypes bearing the same name were assigned to different genetic clusters. Based on a clustering approach, two core sets were constructed. The first core set (Core15, 5.7% of the collection) of 9 *officinalis* and 6 *altissima* genotypes aimed to comprise the diversity of the entire collection with minimal redundancy for long-term breeding programs. An extended core set of 30 genotypes (Core30, 11.3%) focused on the subspecies *officinalis* and included more cultivated material.

**Conclusions:**

This study provides a genomic characterization of a comprehensive *M. officinalis* collection. The identification of distinct genetic clusters and the development of optimized core sets significantly reduce the resources required for future evaluations. These core sets serve as a robust foundation for breeding programs aimed at enhancing agronomic traits such as drought tolerance, while increasing pharmaceutical quality.

**Supplementary Information:**

The online version contains supplementary material available at 10.1186/s12870-026-08853-8.

## Background

Balm (*Melissa officinalis*) is widely used in pharmaceutical products, perfumes, alcoholic preparations, teas and spices, predominantly for its sedative and spasmolytic effects [40]. These effects are attributable to the essential oil and phenolic compounds [33]. *Melissa officinalis* can be divided into at least two subspecies [4]. The usually diploid (2n = 2x = 32 chromosomes) subspecies *Melissa officinalis officinalis* (further short: *officinalis*) harbors the typical lemon aroma, containing the essential oil compounds citronellal, gernial and neral [4]. Therefore, it is also called lemon balm. In contrast, the usually tetraploid (2n = 4x = 64 chromosomes) subspecies *Melissa officinalis altissima* (further short: *altissima*) contains no or very low citral, but germacrene D as major component [32]. Consequently, this subspecies is typically not used for pharmaceutical applications. Nevertheless, *altissima* can be a very interesting gene pool to be used in breeding for agronomic traits and for genetic analyses of important compound biosynthesis. Rarely, a third (diploid) subspecies *Melissa officinalis inodora* is mentioned [4, 41].

Balm is assumed to originate from the Mediterranean region, the Near East and the Caucasian region [51], but due to traditional usage since ancient times it was distributed to whole Europe, Asia and later to North America. From cultivation it was frequently released into the wild [4]. Worldwide, balm is grown as a perennial crop. High initial costs to establish balm into the field, stemming from pre-cultivation in the green house, manual planting and manual weed control, must be amortized by multiple years and harvests within years to gain profit. Therefore, new varieties should harbor strong winter hardiness, good resprouting ability after harvest, as well as high drought and heat stress tolerance to cope with climate change. Moreover, new varieties should contain high contents of pharmaceutical active and aromatic compounds. Additionally, some farmers prefer “erect growth” (found in cultivars like ‘Erfurter Aufrechte’), ensuring a harvest and therefore an income in the year of planting. To compile all these traits in new varieties, suitable genetic resources from multiple origins need to be identified.

Several studies analyzed content and composition of volatile or non-volatile compounds of collections from 10 to 78 accessions, including international accessions from gene banks, botanical gardens and companies [5, 32, 33, 61] or regional wild populations [1, 60]. Some studies included agronomic and morphological traits [60, 61]. Bomme et al. [5] investigated winter-hardiness as well. However, disparate locations, years, and methods, along with few overlaps between accessions, complicate the integration of these studies for comprehensive breeding selections. In addition, a large portion of available international genetic resources remains untested. Thus, a comprehensive study on various traits of a high number of genetic resources is necessary as a solid foundation for an ambitious breeding program. The key limitation, however, lies in the high costs for chemical analyses, extensive personnel requirements for large field trials, and limited space in controlled environments for drought stress experiments. These factors severely restrict the number of genotypes that can be phenotyped and chemotyped within a breeding program.

Since costs for genotyping were considerably reduced in recent years, genomic analysis provides a cost- and time-effective initial filter to identify a core set of most diverse genetic resources, which can then be evaluated in more detail. In addition, many agronomic and metabolite traits are strongly influenced by environment and management, so that cross-study comparisons based solely on phenotype can be difficult. Molecular characterization provides a complementary and more stable basis for assessing genetic relatedness, detecting redundancy or mislabeling, and guiding the selection of genetically diverse subsets [8, 9].

So far, genomic analysis in balm has been limited to 10 until 21 predominately-wild Iranian balm accessions using various marker systems like amplified fragment length polymorphism (AFLP) or start codon targeted (SCoT) markers [11, 24, 35, 55]. In contrast to these fragment-based systems, next generation sequencing (NGS) methods like genotyping by sequencing (GBS) provide high-density, co-dominant SNP datasets that enable more precise estimation of genetic parameters, such as fine-scale population structure. In particular, GBS can provide genome-wide data with high genome coverage, while remaining cost-effective due to genome reduction by enzymatic restriction [18]. For instance, GBS has been successfully applied to analyze population structure, genetic diversity, and to define core sets in hazelnut [57], tea [27] or walnut [56]. While sequencing technology was recently used for transcriptome analysis of one genotype [40], it has not yet been applied for large-scale genotyping of a comprehensive collection.

## Material and methods

### Aim of the study

In this study, we utilized flow cytometric and GBS data from a diverse collection of 256 international *Melissa officinalis* genotypes. As access to in situ populations is often restricted by geographical and legal constraints, we assembled this collection primarily from various international gene banks. This approach allowed us to consolidate a broad spectrum of the globally available genetic diversity, ranging from traditional landraces, commercial cultivars and current breeding material to historical accessions of diverse geographical origins, into a single diversity panel. By including such a wide array of materials, the collection serves as a representative proxy for the genetic variability utilized in both historical cultivation and modern breeding. To analyze this resource, we addressed the following key objectives:

1. To determine genome size and ploidy as a prerequisite for interpreting population genomic data. This cytogenetic screening provides the necessary context to discuss genetic diversity parameters (like H_E_ or private alleles) in relation to the observed cytotypes, ensuring that diversity patterns are evaluated with due consideration of potential ploidy-related effects, while providing a comprehensive cytogenetic screening for many previously uncharacterized international genetic resources. To identify the presence and extent of major and minor substructures within the collection and determine whether these substructures are associated with the available meta-data (e.g., origin, subspecies, classification).
2. To determine the existence of untapped genetic diversity that could be harnessed for future breeding.
3. To evaluate the degree of varietal purity within traditional old varieties that have been collected from different seed providers. Under the assumption that a cultivar represents a consistent genetic entity, we predefined varietal purity as the requirement that all genotypes sharing a cultivar name must form a coherent genetic cluster; failure to co-cluster is interpreted as a loss of varietal integrity.
4. To establish the most efficient core sets that maximize genetic diversity while minimizing genetic redundancy, thereby providing a robust selection of core genotypes for future breeding programs and scientific analyses.

### Germplasm

A collection of 256 genotypes of *Melissa officinalis* was assembled from gene banks and research institutes. Most genotypes derived from single seeds, which were randomly selected from an accession stored as seed material at the provider. In some cases (especially for wild accessions), more than one genotype was derived from one accession. In few cases (e.g. triploids), genotypes derived from material propagated as clonal material. Material from the Bavarian State Research Center for Agriculture (LfL) was once provided as clonally propagated material to JKI, but further propagated as seeds from self-fertilization of those clones. For simplicity, we defined the last distributor or owner of an accession as its provider. Table 1 presents each provider, the number of provided accessions and derived genotypes. This strategy of prioritizing the number of accessions over the number of individuals per accession was chosen to maximize the represented geographical and breeding-history breadth of the diversity panel within the given sequencing capacity.

**Table 1:** Used abbreviation (abbrev.) for providers, details on providers and number of genotypes per provider, in brackets number of different accessions (Ac.) genotypes derived from per provider

Provider abbrev	Provider Details	Genotypes (Ac.)
AGES	Austrian Agency for Health and Food Safety, Plant Genetic Resources, Linz, Austria	5 (5)
AGRO	Agroscope, Nyon, Switzerland	5 (5)
CITA	Agri-Food Research and Technology Centre of Aragon. Horticultural Germplasm Bank, Zaragoza, Spain	9 (4)
CRI	Crop Research Institute, Prague, Czech Republic	6 (5)
IPK	Leibniz Institute of Plant Genetics and Crop Plant Research, Gatersleben, Germany	42 (28)
ISR	Israel Gene Bank, Ministry of Agriculture and Rural Development, Agricultural Research Organization, Rishon LeZion, Israel	28 (28)
JKI	Julius Kuehn-Institute, Institute of Federal Research Centre for Cultivated Plants, Institute for Breeding Research on Horticultural Crops, Quedlinburg, Germany	58 (46)
LFL	Bavarian State Research Center for Agriculture, Freising, Germany	71 (62)
NORD	Nordic Genetic Resource Center (NordGen), Alnarp, Sweden	7 (7)
STEI	Institute of Special Crops, Agricultural Research Center Styria, Wies, Austria	5 (5)
VIR	Vavilov Institute of Plant Genetic Resources, Saint Petersburg, Russia	20 (15)
Total		**256 (215)**

**Fig. 1:**
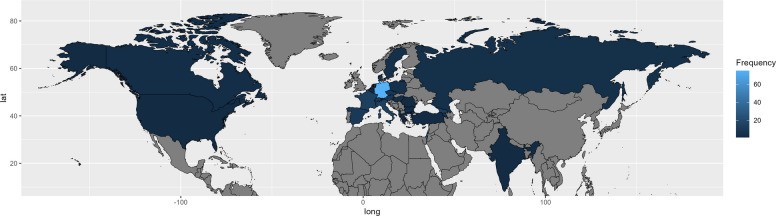
Global Distribution of the Melissa officinalis genotypes (n = 256). Colors: From light blue = high frequency of genotypes to dark blue = low frequency; Grey = No genotypes

Detailed information on each accession is provided in Table S1. Based on passport data and best knowledge, genotypes from these accessions were classified as wild (73), cultivar (41, including land races and unnamed commercial material in general), breeding material (48) or non-classified (94, for accessions not fitting to these categories like accessions with poor passport data or from botanical gardens). Geographical origin was assigned based on passport data. In cases of missing data, the origin was recorded as 'unknown'. For cultivars and advanced breeding lines, the country of the breeder or provider was defined as the origin, unless detailed pedigree records allowed for the identification of the primary genetic source. Based on this definition, the material originated from 27 different countries from the Northern hemisphere. Since most breeding material originated from Germany, Germany is the dominating origin (Fig. 1). Interestingly, 24 breeding lines of the JKI originated from former crosses between accessions from Georgia and France. In general, pedigree information on several breeding lines were useful for internal validation of the used methods and method settings in this study.

### Genome size estimation, ploidy and determination of the subspecies

The genome size of each genotype was estimated using the flow cytometer CytoFLEX (Backman Coulter, Brea, USA) with one measurement per genotype. In cases of low signal quality, measurements were repeated to ensure data integrity. The *Raphanus sativus* cultivar *Saxa* of defined genome size (2C = 1.11 pg) was used as internal reference standard [14, 15]. Stalk fragments of the balm sample and leaf fragments of the reference standard were simultaneously cut with a razor blade. Stalks were used to avoid inhibiting secondary metabolites concentrated in balm leaves. This approach is consistent with established protocols (e.g. [64]), which confirm that various vegetative tissues can be utilized for reliable genome size estimation. The CyStain PI Absolute P reagent kit was used for extraction and staining and CellTrics for filtering (both Sysmex, Kobe, Japan). Ploidy levels were assigned by using the 2 C DNA content as a diagnostic marker, a correlation previously validated for *M. officinalis* via FISH by Kittler et al. [34]. In our study, genotypes formed three discrete, non-overlapping clusters. Diploids (1.69–2.04 pg) and tetraploids (3.38–3.89 pg) showed a clear 1:2 ratio in DNA content, while triploids (approx. 2.71 pg) occupied the expected intermediate position. The substantial gaps between these groups (minimum 0.66 pg) allow for an unambiguous assignment without the need for additional cytogenetic verification for every individual.

**Fig. 2:**
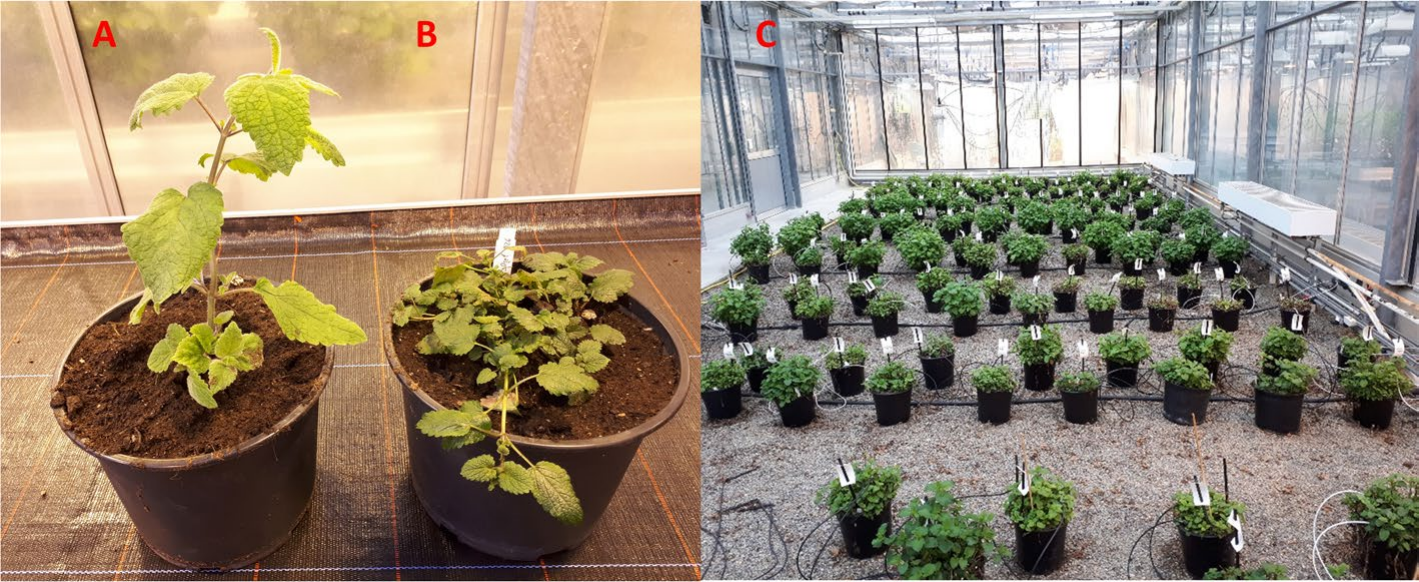
Genotype of the subspecies (A) altissima and (B) officinalis; (C) Part of the balm collection in the green house at the JKI in Quedlinburg

The subspecies of each genotype was simply determined by unambiguous morphological and aromatic characteristics. In contrast to *officinalis*, *altissima* is known to carry very hairy leaves without a lemon-like aroma [51]. Instead, the odor is described as sweet soapy [4]. In addition, *altissima* produce larger and thicker leaves and thicker erected stalks with longer internodes between leaves, which results in clearly different appearance compared to *officinalis* (see Fig. 2).

### Genotyping by sequencing

DNA from young developing leaves was extracted using NucleoSpin Plant II, Mini Kit (Marchery-Nagel, Düren, Germany) applying lysis buffer PL1. DNA quality and quantity were tested using Nanodrop 8000 and Qubit Flex Fluorometer using dsDNA Assay BR (all Thermo Fisher Scientific, Waltham, USA) and gel electrophoresis. Library preparation for GBS was carried out at the Institute of Integrative Biology and Systems (IBIS) of University Laval (Quebec, Canada). Genomic DNAs with a normalized DNA concentration of 10 ng/µl were restricted with the enzymes PstI and MspI [3]. This enzyme combination is widely used to reduce genome complexity by focusing sequencing effort on non-repetitive, gene-rich regions, while ensuring a fragment size distribution suitable for the Illumina platform [52]. The libraries were sequenced with Illumina Novaseq 6000 at Génome Québec (Montreal, Canada) producing150bp paired end reads.

### ‘Mock reference’ and SNP discovery

The platform Galaxy [23] was employed to process raw sequences, constructing a ‘mock reference” and performing mapping to SNP calling. GBS raw reads were trimmed using Trim Galore (v0.6.7; non-default parameters: Phred quality score threshold for low-quality ends: 30 (to ensure high base-calling accuracy); discard reads shorter than 50 nucleotides; [36]).

In absence of an available reference genome, a so-called ‘mock reference’ was built using vsearch clustering (Galaxy version 2.8.3.0; non-default parameter: cluster_fast, identity 0.93, sizein True, sizeout True; [58]). The identity of 0.93 was selected to balance the clustering of polymorphic alleles and the separation of paralogous loci [50]. Beforehand all paired-end reads were merged together using PEAR [70] and dereplicated using vsearch dereplication [58]. Thus, the 'mock reference' was composed of consensus GBS fragments (compare [46]).

Trimmed reads were mapped against the ‘mock reference’ using BWA-mem (Galaxy Version 0.7.18; [37, 38]). SNP calling was carried out using bcftools mpileup and bcftools call (Galaxy Version 1.15.1 + galaxy5; non-default parameters: output-tags DP,DPR; [39]) resulting in a raw VCF file. SNPs were filtered twice: First filter: Minimum quality: 40; minimum number of reads per SNP: 4; Second filter: ≤ 10% missing values, ≥ 5% minor allele frequency (MAF. (to exclude potential sequencing errors and focus on common genetic variation) and ≤ 90% heterozygosity (to prevent the inclusion of collapsed paralogous loci in the ‘mock reference’). Afterwards genotypes with ≥ 20% missing values were discarded. After discarding, the filtering was repeated starting from the unfiltered VCF.

The whole procedure was conducted for the full set of 256 genotypes, as well as for a subset of 202 diploid genotypes of the subspecies *officinalis*.

### Population structure

To assess population structure, principal component analysis (PCA), a Bayesian clustering analysis (STRUCTURE) and a hierarchical agglomerative clustering (HAC) approach were compared. PCA was conducted using the R package adegenet [30, 31]. Bayesian clustering analysis was performed using STRUCTURE v2.3.4 [20, 21, 28, 53]. A putative number of K = 1 to 10 subpopulations was tested within ten independent runs. Markov Chain Monte Carlo (MCMC) iterations and the number of burn-ins were set to 100,000. The output was analyzed using Structure harvester [17]. To select the optimal number of K, the Evanno ∆K method was applied [19].

HAC: The method applied in this study is a minor variation an information retrieval (IR) and natural language processing (NLP) approach. In classical IR and NLP, matrix decomposition has long been used to uncover latent topics in document collections; in our setting, the same principle is applied to identify clusters in genomes represented by their SNP profiles. Prior to decomposition, the term-document matrix (here: SNP-genome matrix) was weighted using term frequency-inverse document frequency (TF-IDF), a standard method to down-weight very frequent but uninformative terms [59]. For dimensionality reduction, we employed latent semantic indexing (LSI), which is well suited for working on the TF-IDF matrix [12]. LSI is based on the singular value decomposition (SVD), which factorizes the matrix into orthogonal components [25]; a truncated version of this factorization captures the dominant latent structure of the data, here rank = 50. To identify clusters within the reduced representations, hierarchical agglomerative clustering (HAC) was applied [29] on a cosine distance and ward.D2 [48] as linkage criterion. The number of clusters (n = 15) was determined by visually inspecting the clustering dendrogram, the data embeddings, and the elbow curve. Cluster assignments were obtained using cutree [54]. To further explore and visualize the reduced representations, we applied uniform manifold approximation and projection (UMAP) [43]. The described methods have been successfully transferred to bioinformatics applications in genomics, both in combination and individually [10, 13, 47]. All preprocessing and analysis steps were carried out in base R [54]. Truncated SVD was computed with the irlba package (version 2.3.5.1; [2]). All visualizations were produced using ggplot2 [69].

In this study, we used the term subpopulation for the first differentiation of the collection and the term (major and minor) cluster for well supported clusters by STRUCTURE and HAC. The term subcluster was merely used to assess within-cluster diversity for constructing the extended core set.

### Genetic diversity

Observed heterozygosity (H_O_) and expected heterozygosity (H_E_) as well as number of unique and common alleles for various subsets were calculated using base R [54].

### Core set construction

Since the HAC approach was found effective in detecting clusters and subclusters of genetic diversity within the balm collection, we utilized this clustering approach to construct two core sets. First, we constructed a small core set (Core15) with high diversity and very low redundancy, including both subspecies. For this purpose, we selected one genotype from ‘the center’ of each of the 15 clusters revealed by HAC (see 2.5 and 3.3.1); in detail via the minimal squared (pairwise) distance within the respected cluster.

Second, we constructed an extended core set (Core30) focusing specifically on the subspecies *officinalis*. To achieve this, the cut-offs of the HAC were relaxed until 20 subclusters were reached within *officinalis*. From each of these subclusters that was not yet represented by a core genotype of Core15, one genotype was chosen. Furthermore, we added four traditional cultivars with known special attributes such as prostrate or erect growth habit (in the first year of cultivation).

## Results

### Genome size estimation, ploidy and determination of the subspecies

**Fig. 3:**
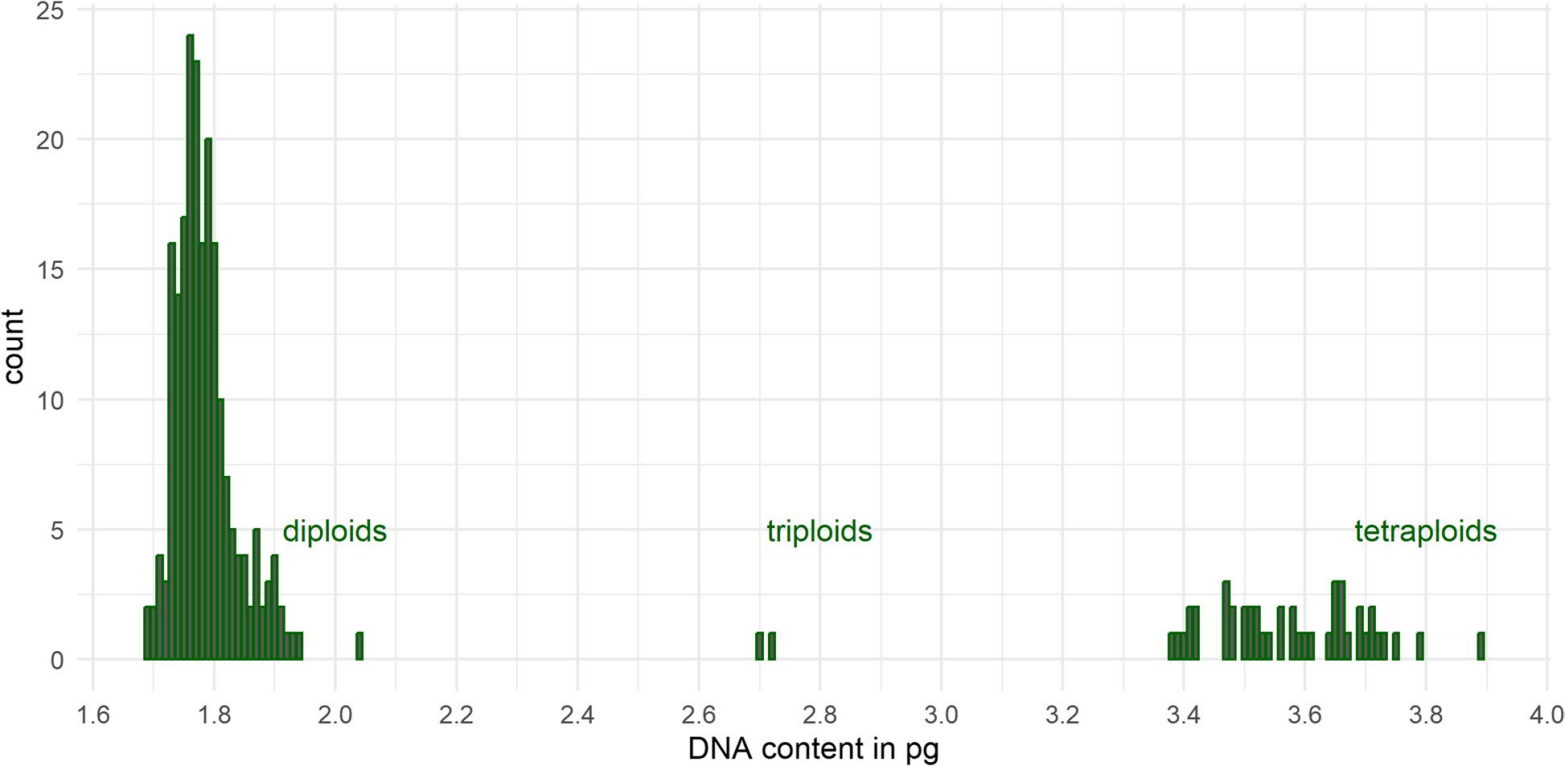
2C/3C/4C DNA content of 256 balm genotypes in pg estimated by flow cytometry. Diploids = 209, triploids = 2, tetraploids = 44

We determined 209 diploid *officinalis* genotypes (on average 2 C = 1.79 pg) and 44 tetraploid *altissima* genotypes (on average 4 C = 3.57 pg). All accessions from the genbank of Israel were determined as *altissima*. In addition, the collection contained 2 triploid (on average 3 C = 2.71 pg) and 1 tetraploid *officinalis* genotype (4C = 3.73 pg). The latter derives from a breeding line of the JKI, which was treated by colchicine to induce tetraploidization. The full range of the detected genome size is depicted in Fig. 3. The coefficient of variation (CV) ranged from 0.02 to 0.05 (on average 0.04) and from 0.02 to 0.06 (on average 0.04) for the reference and the balm sample, respectively (Table S1).

### Raw sequence data, ‘mock reference’ and SNP matrices

The number of raw reads per genotype was on average 8,401,708 and ranged from 432,744 to 18,171,914. The size of the ‘mock references’ and the number of SNPs after different filters for analyzed subsets of genotypes are listed in Table 2. All ‘mock references’ and the SNP matrices are added as supplementary material (Table S2 to Table S5). Large unfiltered VCF files can be made available upon request.

**Table 2:** Number of sequences per ‘mock reference’ (Mock ref.), number of genotypes after filtering and number of SNPs after filtering of different subsets of genotypes

Subset	Sequences ‘Mock ref.’	Genotypes (after filter)	SNPs (1st filter)	SNPs (2nd filter)
All genotypes	79,075	249	85,592	29,307
Diploid *officinalis* genotypes	58,582	196	36,248	9,909

### Population structure & association with meta-data

#### Population structure of 249 officinalis and altissima genotypes based on 29,307 SNPs and association with meta-data

**Fig. 4:**
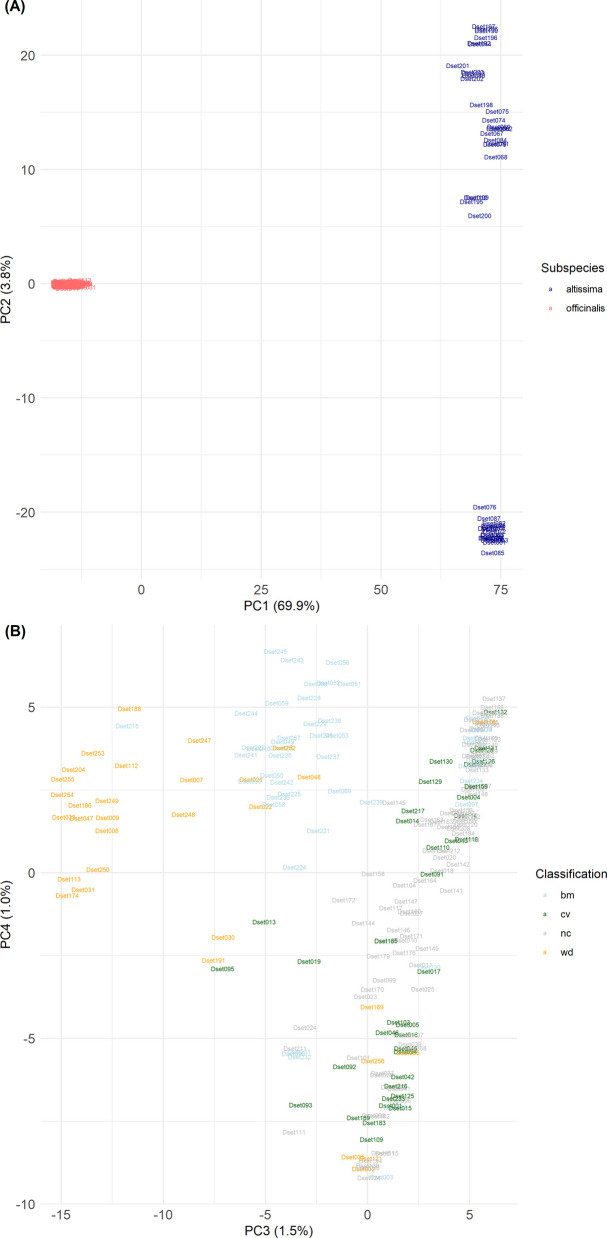
Principal component analysis (PCA) based on 249 genotypes and 29,307 SNPs, presenting (**A**) PC1 and PC2 and (**B**) PC3 and PC4 (displaying the IDs of officinalis genotypes only). The position of each genotype is indicated by the genotype ID (Table S1). PC = Principal component; in brackets explained variance per PC. Colors indicate (**A**) subspecies (red = officinalis; darkblue = altissima) or (**B**) classification (light-blue bm = breeding material, green cv = cultivars and commercial material, grey nc = non-classified material, orange wd = wild)

PCA revealed a strong differentiation into two subpopulations associated with subspecies (Fig. 4A). PC1 explained 69.9% of the variance. PC2 merely explained 3.8% of variance and separated 18 genotypes from Israel and all 26 other *altissima* genotypes (including all 10 other genotypes from Israel). PC3 and PC4 provided resolution within the *officinalis* genotypes (Fig. 4B, *altissima* genotypes are not shown here): PC3, which explained 1.5% of variance, presented some differentiation between wild material (mainly originating from Armenia, France, Georgia and Spain) and the majority of cultivated (93%) and unclassified (100%) genotypes. Breeding material (mainly originating from crosses between material from Georgia and France) appeared to be in between (Fig. 4B). PC4, which explained 1.0% of variance, showed a gradual differentiation within cultivated and unclassified genotypes, which is not clearly associated with other factors like origin or provider (Fig. 4B). More detailed results from PCA can be extracted from Table S1.

**Fig. 5:**
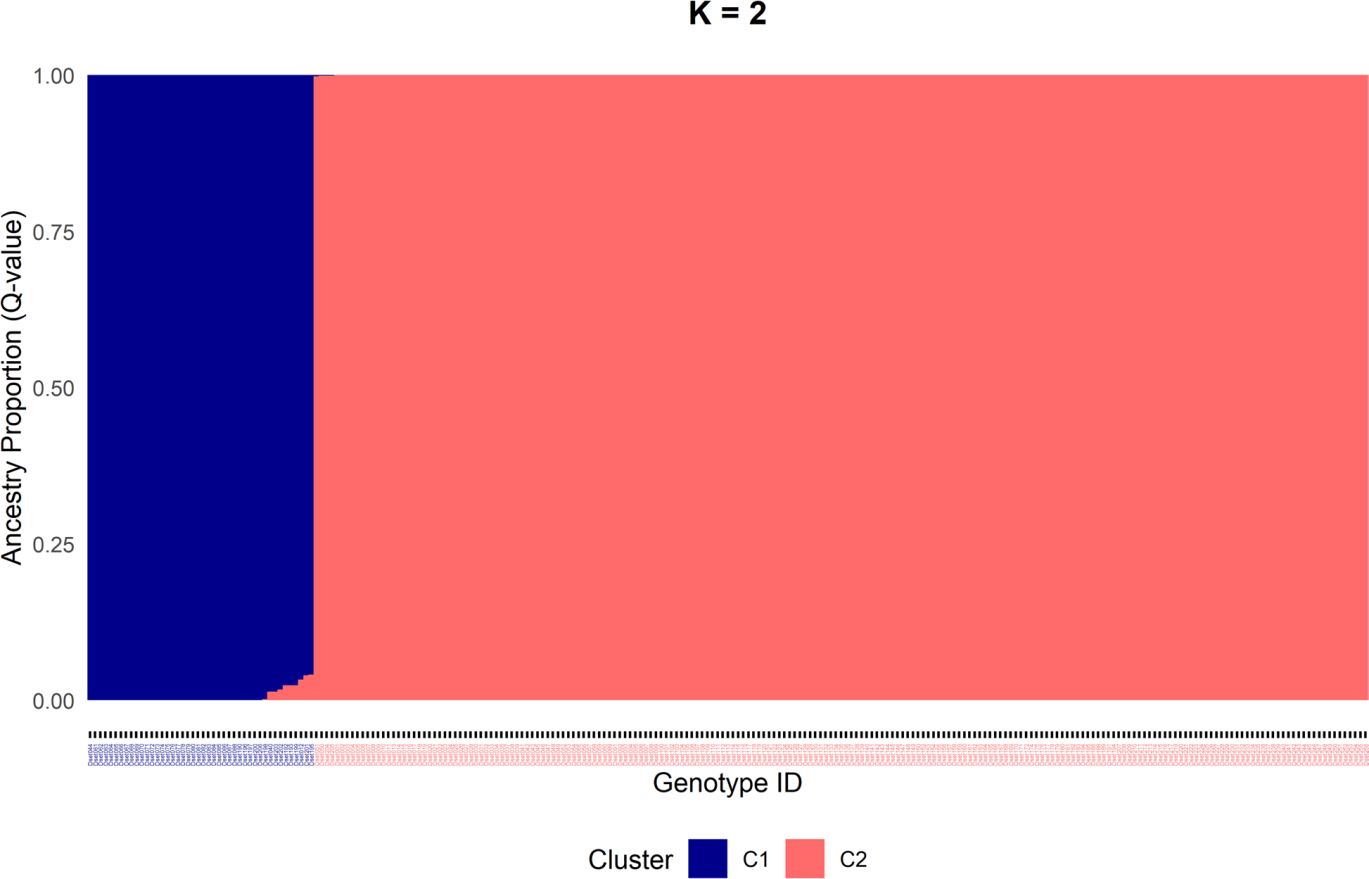
STRUCTURE (Q-values) results based on 249 genotypes and 29,307 SNPs for K = 2. Label colors of genotype-IDs on x-axis indicate subspecies (red = officinalis; darkblue = altissima)

The STRUCTURE results strongly supported the differentiation into two distinct subpopulations (K = 2), clearly separating both subspecies (ΔK = 49,907; Fig. 5, Figure S1). Only a few genotypes exhibited a low level of admixture. ΔK values for other numbers of subpopulations were comparatively very low (at maximum K = 3: ΔK = 42; K = 9: ΔK = 64, Figure S1, Table S1), confirming the two-subpopulation model as the most probable genetic structure.

**Fig. 6:**
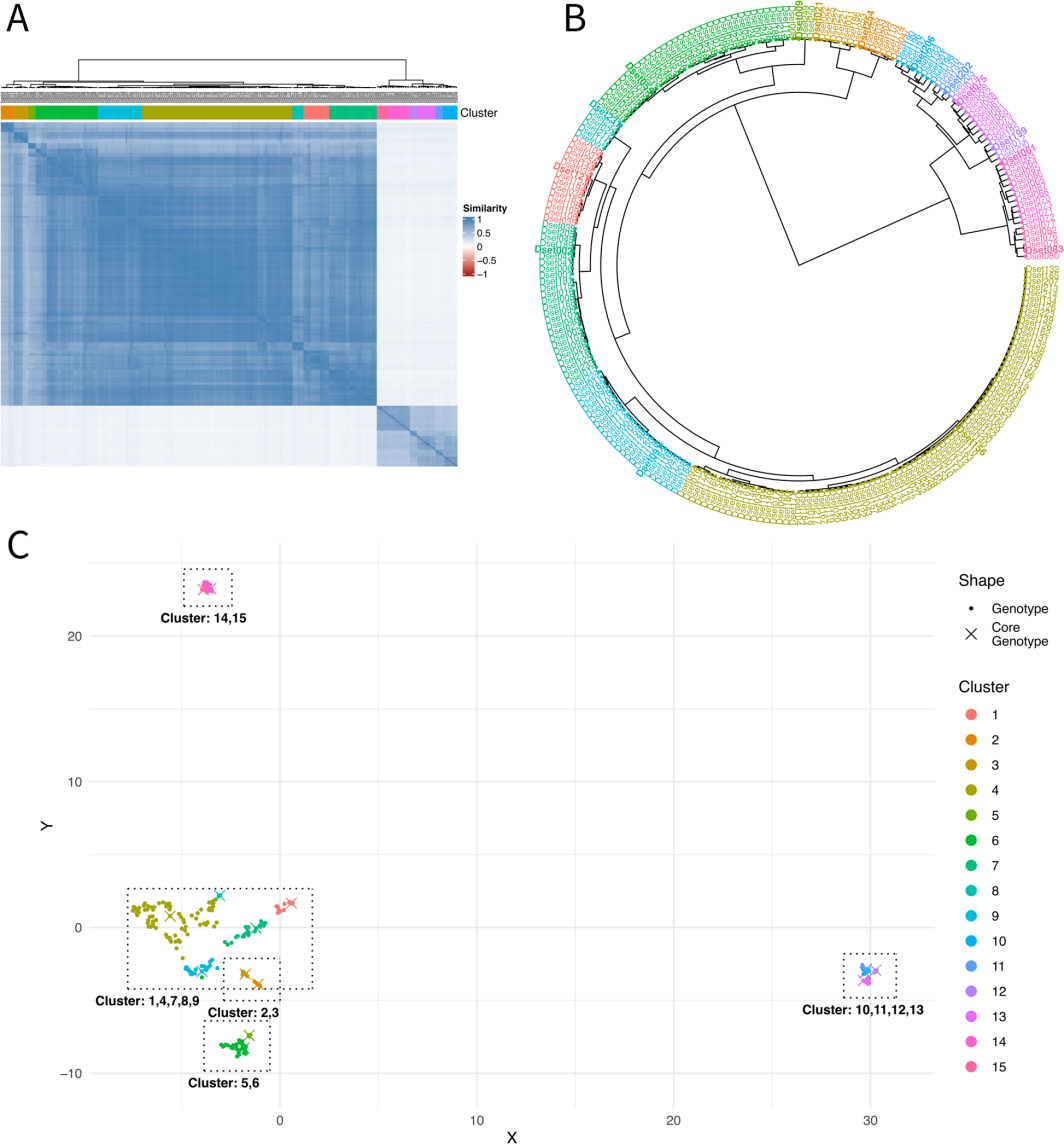
Results from HAC based on 249 genotypes and 29,307 SNPs visualized via multiple perspectives: (**A**) similarity matrix, (**B**) clustering tree and (**C**) 2-dimensional embedding of UMAP. Selected core genotypes (Core15) are highlighted and clusters with comparable properties are grouped with bounding boxes

**Fig. 7:**
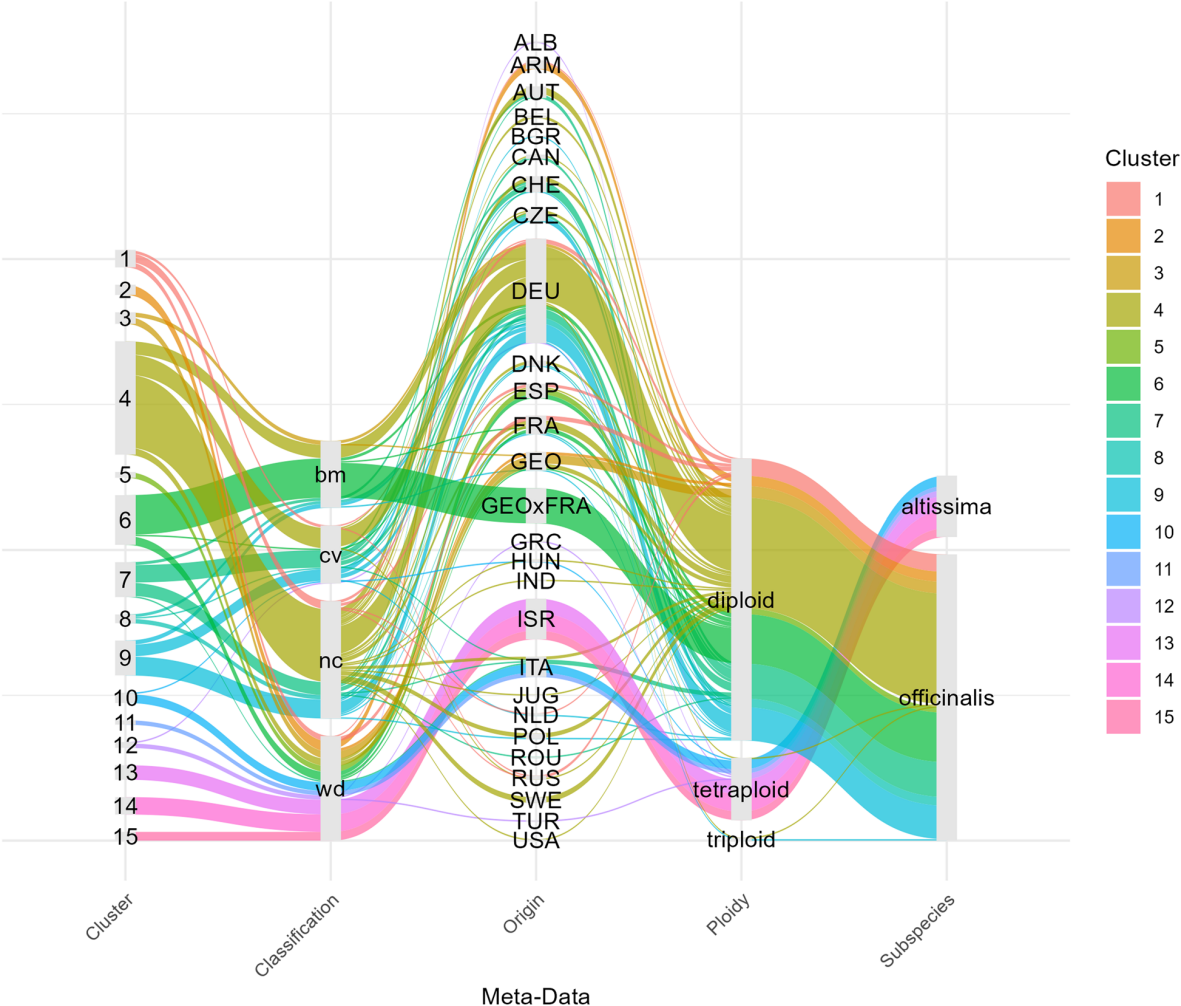
Clusters revealed by HAC based on 249 genotypes and 29,307 SNPs and main attributes of clusters regarding meta-data. Clear paths indicate that clusters are highly descriptive and discriminative in respect to their meta-data attributes

Similar to PCA and STRUCTURE, HAC revealed two major subpopulations associated with subspecies. Moreover, nine and six clusters were detected within *officinalis* and *altissima*, respectively (Fig. 6). Table 3 and Fig. 7 summarize the attributes of these clusters concerning subspecies, classification, and origin: Clusters of *altissima* are strictly separated by origin. Similar to the PCA (Fig. 4A, PC2), cluster 13, composed of Israeli genotypes, was more closely related to the clusters containing genotypes from Italy, Greece, Turkey, and Albania (clusters 10, 11, 12) than to the other two clusters (14, 15) of Israeli genotypes. As a tendency, genotypes of clusters 10 to 13 had a slightly higher genome size (ranging from 3.63 to 3.75 pg) than genotypes of clusters 14 and 15 (ranging from 3.45 to 3.49 pg; Figure S2). Within *officinalis*, five clusters were identified containing most cultivars and non-classified genotypes. Additionally, four distinct clusters were detected containing wild genotypes and breeding material from Georgia/Armenia (Clusters 2 and 3), Spain (Cluster 5), and France (including genotypes derived from crosses between Georgian and French material, Cluster 6).

**Table 3:** Clusters revealed by HAC and main attributes of clusters regarding meta-data (disregarding few exceptions; detailed explanations in Table S1) based on 249 genotypes and 29,307 SNPs. The HAC results are compared with the clusters revealed by STRUCTURE (K = 7) based on 196 genotypes and 9,909 SNPs. Classifications: bm = breeding material, cv = cultivars and commercial material, nc = non-classified material, wd = wild. Origin: Countries based on ISO3166. NA = not applicable

Clusters HAC	Subspecies	Classification	Origin	Clusters STRUCTURE (K = 7)
1, 4, 7, 8, 9	*officinalis*	cv, nc, (bm, wd)	Europe & more	1, 2, 3, 4, 7
2, 3	*officinalis*	wd, (bm)	ARM, GEO	5
5	*officinalis*	wd	ESP	5
6	*officinalis*	bm, wd	GEO x FRA, FRA	6
10, 11	*altissima*	wd	ITA	NA
12	*altissima*	wd	TUR, ALB, GRC	NA
13	*altissima*	wd	ISR	NA
14, 15	*altissima*	wd	ISR	NA

#### Population structure of 196 officinalis genotypes based on 9,909 SNPs and association with meta-data

**Fig. 8:**
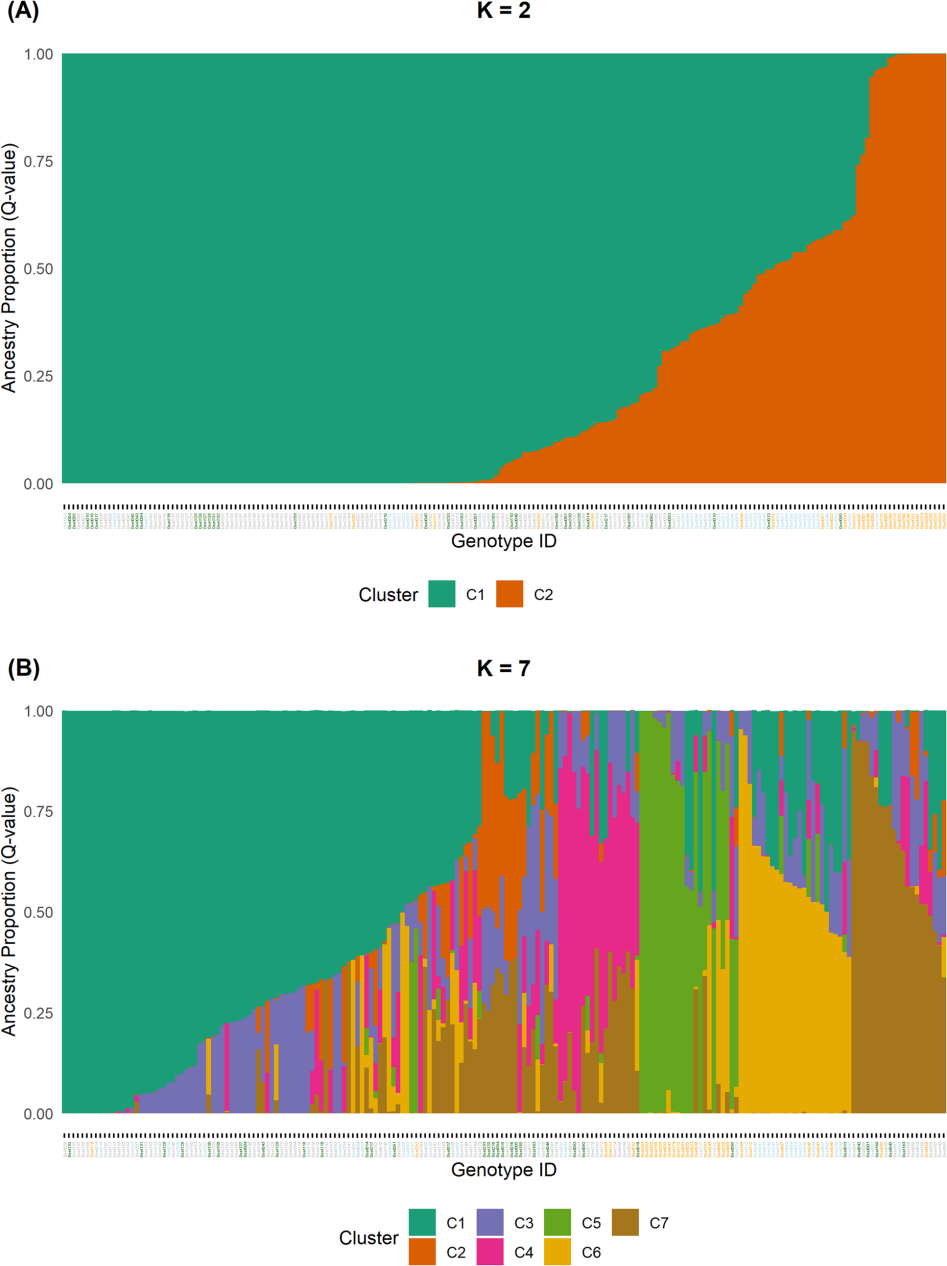
STRUCTURE (Q-values) results based on 196 genotypes and 9,909 SNPs for (**A**) K = 2 and (B) K = 7. Label colors of genotype-IDs on x-axis indicate classification (light-blue = breeding material, green = cultivars and commercial material, grey = non-classified material, orange = wild)

In contrast to PCA and HAC, STRUCTURE did not disclose differentiation within *officinalis* based on the dataset of 29,307 SNPs. Therefore, we performed a second STRUCTURE analysis on the subset of 196 *officinalis* genotypes based on 9,909 SNPs. The Evanno method supported the differentiation into two (ΔK = 766.28), three (ΔK = 129.27) or seven (ΔK = 229.94) clusters (Figure S3, Table S1). The K = 2 model primarily emphasized the differentiation between wild material and breeding material (Cluster 2) versus the majority of cultivated and unclassified genotypes (Cluster 1, Fig. 8A). This result was comparable to the findings of PCA (Fig. 4, PC3) and HAC (Fig. 6).

The K = 7 model revealed five (minor) clusters within the cultivated and unclassified genotypes, a pattern similar to the five corresponding clusters identified by HAC (Table 3). However, the specific assignment of genotypes to clusters was not fully identical between STRUCTURE and HAC. Figure 8B also displays a high proportion of admixture in these clusters, particularly in clusters 2 and 3 (mean membership coefficient: 0.40 and 0.36, respectively). Furthermore, the (major) cluster containing wild material and breeding material was divided into only two (minor) clusters based on STRUCTURE results, rather than the four (minor) clusters detected by HAC. Specifically, the differentiation between genotypes from Georgia/Armenia and Spain is not supported by the STRUCTURE K = 7 model (Table 3).

In addition, we compared the PCA based on the full dataset of 29,307 SNPs with the PCA based on the subset of 9,909 SNPs. Briefly, the latter PCA (9,909 SNPs) revealed a low population structure, where the explained variance was merely 6.9% for PC1 and 4.3% for PC2 (Figure S4A). Importantly, the resulting differentiation pattern was highly similar to the gradual differentiation revealed by PC3 and PC4 in the PCA of the full dataset (29,307 SNPs) (Fig. 4B).

### Degree of varietal purity among traditional old varieties

We analyzed whether genotypes from different providers carrying the same cultivar name clustered together when analyzed by HAC based on 29,307 SNPs and STRUCTURE (K = 2 and K = 7). Specifically, the four old traditional cultivars *Quedlinburger Niederliegende, Lorelei, Erfurter Aufrechte*, and *Citra* were investigated. For all cultivars except *Lorelei*, genotypes were attributed to different clusters in HAC and STRUCTURE (K = 7, Table 4). The two genotypes of *Quedlinburger Niederliegende* even appeared in different clusters in STRUCTURE (K = 2), indicating a significant genetic divergence. The genetic distance between cultivars of the same name is also visually discernible in PCA (Figure S4B highlights the position of cultivars).

**Table 4:** Number of genotypes per cultivar name and clusters of these genotypes (in brackets number of genotypes in these clusters) detected by HAC based on 249 genotypes and 29,307 SNPs and STRUCTURE (K = 2 and K = 7) based on 196 genotypes and 9,909 SNPs. Cluster numeration per analysis is random

Cultivar name	Number of genotypes	Clusters in HAC	Clusters in STRUCTURE (K = 2)	Clusters in STRUCTURE (K = 7)
Citra	5	C4 (1), C7 (2) C9 (2)	C1 (5)	C1 (2), C2 (1), C3 (1), C7 (1)
Erfurter Aufrechte	4	C1 (1), C4 (2), C9 (1)	C1 (4)	C1 (1), C3 (1), C4 (1), C7 (1)
Lorelei	3	C7 (3)	C1 (3)	C2 (3)
Quedlinburger Niederliegende	2	C4 (1), C9 (1)	C1 (1), C2 (1)	C1 (1), C5 (1)

### Genetic diversity

#### Genetic diversity of 249 genotypes in relation to the subspecies based on 29,307 SNPs

**Fig. 9:**
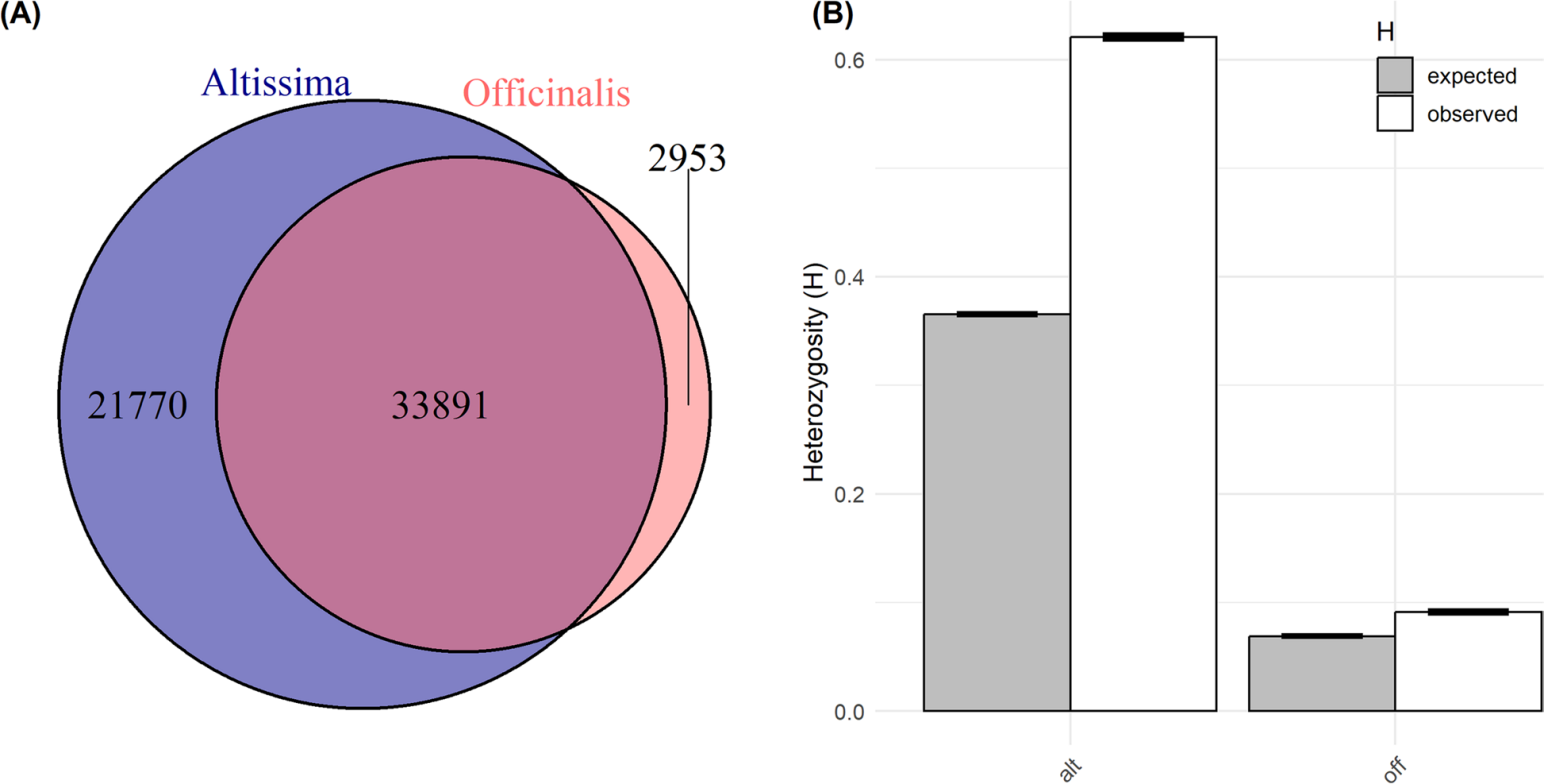
**A** Number of common and private alleles among subspecies. **B** Expected and observed heterozygosity of subspecies. Both based on 249 genotypes and 29,307 SNPs

Since the population structure of the entire collection was strongly associated with subspecies, we compared H_E_ and the number of private alleles as measurements of genetic diversity between both subspecies. Out of a total of 58,614 alleles, *altissima* possessed 21,770 private alleles, while *officinalis* possessed 2,953 private alleles (Fig. 9A). Consequently, both H_O_ and H_E_ were significantly higher in *altissima* (0.62 and 0.38 respectively, Fig. 9B; paired t-test across loci: p < 0.001) compared to *officinalis* (0.09 and 0.07, respectively).

#### Genetic diversity of 196 officinalis genotypes in relation to the classification based on 9,909 SNPs

**Fig. 10:**
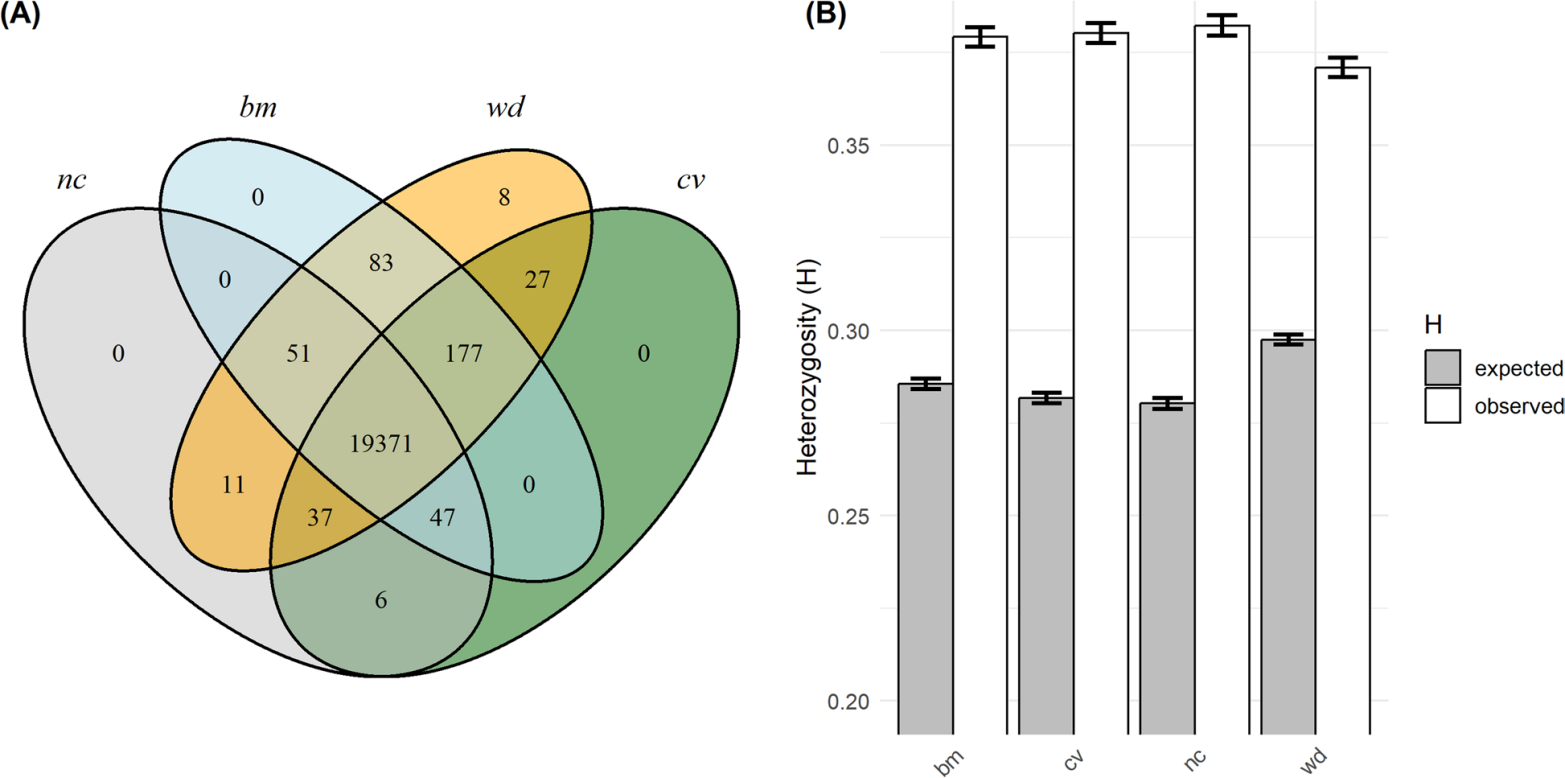
**A** Number of common and private alleles among classifications. **B** Expected and observed heterozygosity of classifications. Both based on 196 genotypes and 9,909 SNPs. Colors indicate classification (light-blue bm = breeding material, green cv = cultivars and commercial material, grey nc = non-classified material, orange wd = wild)

Within the *officinalis* subset we were mainly interested in the potential of non-cultivated material to extend genetic diversity in lemon balm. Therefore, we compared H_E_ and number of private alleles between the different classifications. A relatively low number of private alleles—only 153 alleles—were found among all three non-cultivated accessions (breeding material, non-classified and wild), while 19,371 alleles were common among all classifications (Fig. 10A). Despite this low number of private alleles, H_E_ was significantly higher for wild genotypes compared to other classifications (Fig. 10B; paired t-tests across loci: p < 0.001, differences from 0.012 to 0.016). Conspicuously, H_O_ calculated for the *officinalis* accessions based on the subset analysis alone was considerably higher than when these accessions were analyzed together with *altissima* (Figs. 9B and 10B).

### Core set construction

The core genotypes selected for the Core15 and the extended core set Core30 are listed in Table 5, including their respective meta-data. The core sets are strongly dominated by wild material, which originates primarily from the assumed centers of diversity, including the Mediterranean region, the Near East, and the Caucasian region. Regarding breeding material and cultivars, most material originates from Germany and Switzerland.

**Table 5:** Core genotypes of the core set Core15 and the extended core set Core30 and meta-data. Classifications: bm = breeding material, cv = cultivars and commercial material, nc = non-classified material, wd = wild. Origin: Country abbreviations based on ISO3166

Genotype ID	Subspecies	Ploidy	Classification	Origin	Cultivar
Core15					
Dset121	*officinalis*	diploid	wd	FRA	
Dset254	*officinalis*	diploid	wd	ARM	
Dset221	*officinalis*	diploid	bm	DEU	
Dset156	*officinalis*	diploid	nc	DEU	
Dset009	*officinalis*	diploid	wd	ESP	
Dset055	*officinalis*	diploid	bm	GEOxFRA	
Dset002	*officinalis*	diploid	bm	CHE	
Dset231	*officinalis*	diploid	bm	DEU	
Dset091	*officinalis*	diploid	cv	CZE	Citra
Dset206	*altissima*	tetraploid	wd	ITA	
Dset202	*altissima*	tetraploid	wd	ITA	
Dset199	*altissima*	tetraploid	wd	ALB	
Dset075	*altissima*	tetraploid	wd	ISR	
Dset061	*altissima*	tetraploid	wd	ISR	
Dset083	*altissima*	tetraploid	wd	ISR	
Extension to Core30					
Dset256	*officinalis*	diploid	wd	FRA	
Dset047	*officinalis*	diploid	wd	GEO	
Dset021	*officinalis*	diploid	wd	GEO	
Dset186	*officinalis*	diploid	wd	GEO	
Dset020	*officinalis*	diploid	nc	POL	
Dset210	*officinalis*	diploid	nc	SWE	
Dset092	*officinalis*	diploid	cv	DEU	Erfurter Aufrechte
Dset188	*officinalis*	diploid	wd	FRA	
Dset248	*officinalis*	diploid	wd	ESP	
Dset096	*officinalis*	diploid	bm	DEU	
Dset057	*officinalis*	diploid	bm	GEOxFRA	
Dset233	*officinalis*	diploid	cv	DEU	Citronella
Dset005	*officinalis*	diploid	cv	CHE	Lorelei
Dset095	*officinalis*	diploid	cv	DEU	Quedlinburger Niederliegende
Dset105	*officinalis*	diploid	nc	FRA	

## Discussion

### Limitations of the collection and the study design

While this international collection sourced from gene banks and institutes represents only a fraction of the global genetic reservoir of *Melissa officinalis*, its composition—with a deliberate focus on Central European breeding materials—was chosen to prioritize the germplasm most relevant to current research and international breeding programs.. Numerous natural populations exist from the Caucasian region to the Iberian Peninsula that have never been sampled by gene banks or utilized by breeders. Therefore, this study can only outline hotspots of genetic diversity among those genotypes that are currently accessible to international research and breeding programs. This biased and uneven sampling of the germplasm must be acknowledged when discussing population structure and, particularly, when evaluating measurements of genetic diversity. Nevertheless, the distinct genetic signals observed, such as the dominance of small groups of wild accessions in clustering analyses despite their underrepresentation, underscore the robustness of the identified diversity patterns even within this biased sampling framework.We chose to analyze single individual plants per accession. While an individual plant may largely represent an accession in the case of inbred lines, it only represents a random fraction of the intra-accession diversity in population cultivars or wild accessions. To better capture the genetic diversity, particularly in wild accessions originating from the assumed centers of diversity (the Mediterranean region, the Near East, and the Caucasian region), we analyzed multiple individual plants per these accessions. (If our data are used, e.g. for marker design, we may recommend to search for multiple individuals per accessions via the provider ID in Table S1). Nevertheless, it must be considered that our data cannot fully represent international accessions exhibiting high heterozygosity.

For breeding purposes, we recommend utilizing multiple plants from the identified accessions/gene pools of untapped genetic diversity. This strategy increases the probability of capturing favorable alleles from these gene pools. Crucially, this study cannot make definitive statements on the actual breeding potential of any gene pool regarding specific breeding targets; this needs to be addressed in further phenotypic and chemotypic evaluations.

### Genome size estimation, ploidy and determination of the subspecies

Genome size was estimated by flow cytometry using an internal standard, which is regarded as best practice for genome size estimation [14]. Due to potentially highly concentrated staining inhibitors in balm leaves, a major challenge in flow cytometry [26], we used stalks in this study. Zonneveld [72] states a genome size of 2 C = 1.94 pg for one balm genotype, which is consistent with the range observed in our results for diploid balm (Fig. 3). To the best of our knowledge other published studies on genome size in balm; therefore, this study provides novel and comprehensive data on the species' genome size.

*Raphanus sativus* was chosen as the standard because its peak did not interfere with any *Melissa officinalis* peak. However, the distance between the *R. sativus* peak and the peak of tetraploid balm is larger than the distance to the diploid peak. This may contribute to the slightly higher variance observed in the genome size of tetraploid balm genotypes (Fig. 2), meaning the observed range from 3.4 to 3.9 pg may not necessarily be attributable to real biological variability.

Nevertheless, precise estimation of genome size was not the major goal of flow cytometric analysis but rather the prediction of the ploidy level. Kittler et al. [34] have shown by fluorescent in situ hybridization (FISH) using 18/25S and 5S rDNA that ploidy in balm can be predicted based on flow cytometric results. Our findings that most *altissima* genotypes are tetraploid and most *officinalis* genotypes are diploid are in accordance with the literature [4, 34]. One tetraploid *officinalis* genotype from the JKI has been produced by colchicine treatment. We further determined two triploid genotypes as *officinalis* genotypes. To our knowledge, both genotypes were artificially produced in a breeding program. In contrast, Kittler et al. [34] described four ‘natural” triploid genotypes belonging to *altissima*, which were not available for this study.

### ‘Mock reference’ and diploidized SNP calling

Currently no reference genome for *Melissa officinalis* is available. Consequently, a de novo approach using a ‘mock reference’ consisting of small contigs in random order was necessary. The absence of a reference genome can aggravate inherent issues of GBS, such as pseudo-heterozygosity [44]. Nevertheless, GBS or similar NGS based methods has proven to be a robust method to generate genome-wide marker sets for population genetic studies, even in species without reference genome including several medicinal plant species [16, 49, 67].

We compared diploids and tetraploids using a simplified diploid calling for all genotypes, which is a frequently used approach in diversity studies (examples using NGS data: [62, 68]). A more complex ‘ploidy-aware’ SNP calling, which would allow for the assessment of allele dosages, would require a substantially higher sequencing depth [3, 42]. Therefore, in the context of this study, the diploid calling approach allowed us to sequence a relatively high number of 256 genotypes at low costs. However, for discussing the differences between diploid *officinalis* and tetraploid *altissima* this simplified approach should to be treated with caution, in particular concerning heterozygosity measurements [44].

### Population structure & association with meta-data

#### Choice of methods to assess population structure

To assess population structure, we compared three complementary methods, each offering distinct advantages for analyzing our plant material. PCA and STRUCTURE proved robust for detecting major subpopulations, with PCA providing a rapid, model-free overview of the primary genetic gradients. In addition, STRUCTURE provided critical information on admixture. This model-based approach is further supported by HAC, which independently validates the identified groups through hierarchical distances, without being tied to specific population genetic assumptions. While STRUCTURE relativizes strict divisions into discrete clusters, HAC proved particularly effective for detecting fine-scaled subclusters, making it a robust tool for core set construction. The convergence of these three methods ensures that the inferred genetic structure reflects a consistent biological pattern rather than an algorithmic artifact.

#### Population structure associated with subspecies

PCA, STRUCTURE, and HAC consistently supported the separation into two distinct major subpopulations associated with the respective subspecies (Fig. 4, 5 and 6). This strong differentiation aligns with the observed difference in ploidy level. Indeed, the ploidy difference likely explains this separation, as it may pose a strict crossing barrier between the two subspecies, even in areas where they co-occur. To our knowledge, no other studies have compared *officinalis* and *altissima* genotypes based on genetic markers. However, some studies have revealed a similarly strong differentiation between the two subspecies based on the composition of their essential oils [7, 33]. We will further discuss both subpopulations under the topic of untapped genetic diversity.

#### Clusters within altissima

PCA and HAC consistently supported a clear differentiation of the Israeli *altissima* genotypes into two clusters. Intriguingly, Israeli genotypes of cluster 13 were closer related to European *altissima* genotypes (Figs. 4A, and 6, Table 3) than to Israeli cluster 14 and 15. Since, Israel is a rather small country, and collection sites are relatively close; we cannot propose a geographic crossing barrier within Israel (even stronger than the distance to Italy) that could explain these findings. Interestingly, Israeli clusters tend to differ in genome size (Figure S3). Therefore, it could be relevant for further research to compare the chromosomal organization of these Israeli clusters to investigate potential structural genomic differences, which also might be a reason for a crossing barrier.

#### Clusters within officinalis

First of all, PCA, STRUCTURE, and HAC consistently supported the differentiation of *officinalis* into two (major) clusters, separating a large group of cultivated and non-classified genotypes and few wild genotypes from the majority of wild genotypes and breeding material (Figs. 4B and 8A, Fig. 6).

Looking into (minor) clusters within these (major) clusters provides more interesting insights into the diversity of *officinalis*. While HAC detected nine clusters within *officinalis*, STRUCTURE supported seven clusters. The primary difference was that STRUCTURE grouped the HAC clusters 2, 3, and 5, which contain wild genotypes from Armenia, Georgia, and Spain, into a single cluster (Table 3). This variation can simply be explained by the differing cut-offs used by the methods to define clusters. Nevertheless, the nearly perfect alignment of HAC clusters 2, 3, 5, and 6 to certain geographic origins strongly supports the existence of these finer genetic structures (Table 3).

Within the five clusters (HAC: 1, 4, 7, 8, 9; STRUCTURE: 1, 2, 3, 4, 7; Table 3) comprising European cultivated and non-classified genotypes, STRUCTURE partially revealed high admixture (Fig. 8B), which corresponds to the gradual differences observed in PCA (Fig. 4B). Consequently, the strict assignment of genotypes to clusters based solely on the highest Q-value is a simplification. This diffuse structure might be the reason for partially deviating assignments of genotypes between STRUCTURE and HAC in this group. Furthermore, we could not align these clusters to distinct origins, which could have been expected based on specialized national breeding programs. This lack of clear differentiation is likely explained by active European material exchange for breeding and production and/or by a historically low level of selection intensity in European breeding efforts.

We will further discuss the clusters of *officinalis* under the topic of untapped genetic diversity.

### Degree of varietal purity among traditional old varieties

There are a few ‘famous’ traditional cultivars of lemon balm, whose names can be found in several gene banks, institutes and companies. Since most traditional cultivars have not been subject to variety protection for a long time, they have been propagated by numerous seed producers. It was suspected that seed batches carrying the same cultivar name no longer represent the same cultivar, indicating a loss of varietal purity across seed producers. Initial hints of this issue arose from a field trial in Switzerland, where plants marketed as *Quedlinburger Niederliegende* (‘niederliegend’ = German for ‘prostrate’) exhibited an erect growth habit [63]. Furthermore, the gene bank of the Steiermark indicated the presence of both prostrate and erect types within the cultivar Citra. In terms of varietal homogeneity, two distinct growth types would be unacceptable. In general, the situation of varietal protection in balm (as is the case in numerous medicinal plant species) is significantly different from that in major crops. In major crops, varietal purity can often be reliably checked using established phenotypic and genetic methods [66].

Since traditional cultivars are typically population cultivars Simonnet et al. [63] states that Lorelei is a synthetic cultivar), we did not assume that all analyzed genotypes from the same cultivar should be genetically identical, but rather that all genotypes should at least fall within the same genetic cluster due to the selection process. Table 4 shows that this was not the case for *Quedlinburger Niederliegende*, *Erfurter Aufrechte*, and *Citra*, but it was true for *Lorelei*. Thus, we confirmed a loss of varietal purity across different providers for three of the four traditional cultivars investigated. Consequently, farmers should not rely solely on cultivar names but must check the quality of seed batches for critical traits. Although we cannot determine which genotypes are nearest to the original traditional cultivar based on the genetic data alone, the standard properties of a cultivar can be checked through field trials. Taking advantage of this situation, the diversity discovered between seed lots sharing the same cultivar name can be considered a pool of useful genetic variability for subsequent breeding efforts.

### Untapped genetic diversity

Simplified, all major and minor detected clusters that are devoid of commercial material may be considered gene pools of untapped genetic diversity. These untapped gene pools may harbor high potential for novel alleles for further improving traits, such as increasing essential oil content, winter-hardiness, drought or heat tolerance, or pest resistances. While these clusters represent significant genomic potential, it must be noted that in the absence of comprehensive phenotypic data, the actual breeding value of these specific accessions remains to be validated in subsequent field trials.

Since nearly all *altissima* genotypes are classified as wild, this entire subpopulation represents the largest gene pool of untapped genetic diversity in balm. The high H_E_ measurements hint at a much higher genetic diversity within the *altissima* subpopulation. Moreover, the high number of unique alleles within *altissima* and the relatively high number of clusters within this small *altissima* set clearly emphasize its high genetic diversity compared to *officinalis* (Fig. 9).

Of course, biases resulting from ploidy must be discussed: Since the simplified diploid calling (see 4.3) does not consider allele dosage (AAAa, AAaa, and Aaaa are all considered as Aa), this have caused biased H_E_ results, to some degree inflating heterozygosity in the tetraploids [45]. According to the findings by Meirmans et al. [45], this lack of dosage information can lead to an overestimation of H_E_ by approximately 10–15%. However, this technical inflation cannot account for the five-fold difference observed between the subspecies H_E_ = 0.38 for altissima vs. 0.07 for officinalis).. While comparing genotypes of different ploidies might generally be regarded as ‘unfair,’ the results for *altissima* may simply reflect the 'evolutionary advantage' of polyploids. Polyploidization is a key factor for diversification in plants by increasing the probability of mutations [45], allowing for gene neo-functionalization [71], and enabling genome re-organization [65]. In addition to ploidy, the sampling strategy likely contributes to the high diversity metrics observed across all classifications. Since our collection comprises genotypes from broad geographic origins and diverse ecotypes, the results reflect the aggregate diversity of the entire subspecies' gene pool rather than the internal structure of localized populations. This extensive sampling ensures that rare alleles from isolated locations are captured, which naturally leads to higher H_O_ and H_E_ values compared to studies focused on narrow regional populations. Nevertheless, these higher diversity metrics should be interpreted with caution, as the direct correlation between these genomic indices and superior agronomic performance is yet to be established through systematic trait evaluation.

While the bias from ploidy remains a not fully resolvable issue for diversity measures, the crossing barrier imposed by ploidy presents a more severe technical challenge for utilizing *altissima* in balm breeding. Artificially, crosses between *altissima* and *officinalis* can be conducted but typically result in male-sterile triploids. To overcome this, two strategies are possible: (A) Breeding can utilize tetraploid *officinalis*, potentially generated through colchicine treatment (as done for genotype Dset234). This strategy accepts the challenges inherent to breeding in tetraploids, such as the fixation of trait characteristics due to having four alleles per locus. (B) Triploids from crossing diploid with tetraploid might be reduced to diploids through backcrosses utilizing embryo rescue techniques requiring good laboratory skills. Independent of the chosen strategy, the characteristic lemon aroma of *officinalis* must be re-established for most commercial applications. Thus, breeding with *altissima* unavoidably implies a longer and more resource-intensive breeding process than breeding exclusively within diploid *officinalis*, but it can be worthwhile due to the high number of unique alleles already detected for the reduced genomic representation from GBS (Fig. 9).

Nevertheless, medium-term breeding programs are likely to focus on the genetic diversity within *officinalis*. For this purpose, we detected several clusters containing wild material originating from Armenia, France, Georgia and Spain, which aligns with the assumed centers of genetic diversity. To our knowledge, these gene pools have not been previously described in literature. However, existing crosses between material from France and Georgia (Cluster 6, Table 3) indicate that some value from these clusters had already been identified by breeders. The wild material in general showed a higher H_E_, but a rather low number of unique alleles was detected for non-commercial genotypes for the reduced genomic representation from GBS (Fig. 10). Since the number of genotypes per classification is relatively evenly distributed, this cannot be explained by bias from filter settings. Nevertheless, in addition to integrating novel alleles, breeding also can profit from novel allele combinations across linked loci (haplotypes), which might indirectly be displayed by the divergent clusters identified in this study.

Besides the discussion on untapped genetic diversity, we should take into account that there is also significant genetic diversity within the gene pool of cultivated material. In PCA, this is displayed by a gradual differentiation (Fig. 4B, Figure S4), and in STRUCTURE and HAC by the differentiation into five clusters (Figs. 6 and 8B, Table 3) and partially by high admixture within clusters (Fig. 8B). Moreover, most detected alleles are present in cultivated material (Fig. 10A). The clusters of cultivated material also contain several wild genotypes from France and Spain, as well as all non-classified genotypes (e.g., many genotypes once collected from botanical gardens, Fig. 7). These findings indicate that cultivated material was built upon diverse origins and that breeding has not caused a severe reduction of the gene pool. Anyway, likely applied breeding programs will continue to build upon ‘elite’ cultivars or breeding lines that already contain many favorable alleles from these ‘tapped’ gene pools, which should further be enriched by untapped genetic diversity.

### Core set construction

In general breeders strive to optimally allocate restricted breeding resources. Even more so in minor crops, such as most medicinal plants, where resources for breeding are very limited. Optimizing initial genetic diversity while minimizing genetic redundancy by using a core set can substantially improve the efficiency of a breeding program. For this purpose, we constructed a first small core set of 15 core genotypes (Core15, Table 5) representing each of the 15 clusters detected by HAC. Selecting core genotypes from genetic (or phenotypic) clusters was first proposed by Brown [6]. Although Core15 represents a very small fraction of the entire collection, this number of genotypes might be the optimal selection given certain budget and resource constraints. This first core set is dominated by wild material from clusters of untapped genetic diversity, potentially containing many unused favorable alleles but also disadvantageous alleles for cultivation. Therefore, as discussed above (chapter 4.6), these wild genotypes may rather be used to enrich an existing pool of cultivated material than replacing it.

Since the first small core set merely contained nine genotypes of the economically more important subspecies *officinalis*, we constructed an extended core set of 24 *officinalis* and six *altissima* genotypes (Core30, Table 5). (The six altissima core genotypes might be optional, depending on the specific breeding program or scientific analyses, as challenges for integrating *altissima* are discussed in Chapter 4.6.). Following the idea that the number of chosen genotypes per cluster should depend on the within-cluster diversity [22], we selected additional core genotypes from the best supported subclusters within clusters by HAC. Most subclusters were detected within the clusters of wild genotypes (Table 5), aligning to the higher genetic diversity of wild *officinalis* germplasm. To mitigate the ‘under-representation” of cultivated material in Core30, we deliberately included traditional cultivars that harbor specific attributes such as erect or prostrate growth. We hypothesize that such habits can strongly affect certain traits, like winter-hardiness or drought and heat tolerance, necessitating the inclusion of genotypes with these habits in scientific trials. Considering the issue of varietal purity (Chapter 4.5), we chose genotypes from long-term seed storage as the source for traditional cultivars, as these sources still evidently harbor the required attributes.

Both core sets (Core15 and Core30) should be viewed as recommendations that reflect the current genetic knowledge and the assumed needs for subsequent steps in breeding and breeding research. The comprehensive data provided in this manuscript and its supplements allows breeders and research groups to customize the core sets according to their specialized needs and breeding objective. Further research on specific trait characteristics will likely alter these core sets, which are currently predominantly defined through genetic analysis. So far, there are sparse phenotypic and chemotypic data on some of the investigated accessions [5, 32, 33], but the fact that we sequenced single plants from these accessions, the large gaps of, as well as variance in investigated traits and methodology etc. exacerbated any inclusion of these data.

## Conclusions

The pronounced key objectives of this study were successfully achieved:

1. We determined genome size and ploidy for all genotypes, establishing essential background information for genetic analyses and corroborating a strong association of genome size/ploidy with subspecies.
2. We identified two major subpopulations associated with subspecies. Within these, wild accessions formed several well-defined clusters that aligned closely with their geographic origin. In contrast, European cultivated and non-classified genotypes exhibited a more diffuse structure with high levels of admixture, reflecting the complex breeding history and extensive germplasm exchange in this group.
3. We determined the subspecies *altissima* as one large gene pool of untapped genetic diversity, alongside several clusters of wild *officinalis* genotypes from Armenia, France, Georgia and Spain.
4. We found a loss of varietal purity for three of four traditional cultivars investigated.
5. We established two core sets for balm, providing a robust selection of core genotypes for future breeding programs and scientific analyses.

Further research is needed to disclose whether the identified clusters of genetic diversity, currently defined based on pure genetic analyses, indeed harbor valuable properties regarding key traits of interest, such as winter hardiness, resprouting ability, drought and heat stress tolerance, or high contents of pharmaceutically active and aromatic compounds. The constructed core sets should significantly reduce the costs and resources required for these comprehensive phenotypic and chemotypic evaluations.

## Supplementary Information

Supplementary Material 1: Table S1 Detailed overview of all genotypes of the collection. Detailed results from flow cytometry, PCA, STRUCTURE and HAC.

Supplementary Material 2: Table S2 Filtered SNP matrix of 29,307 SNPs from 249 genotypes. Details in manuscript.

Supplementary Material 3: Table S3 Filtered SNP matrix of 9,909 SNPs from 196 genotypes of subspecies officinalis. Details in manuscript.

Supplementary Material 4: Table S4 ‘Mock reference’ of 79,075 sequences/contigs based on 256 genotypes built by vsearch clustering. Details in manuscript.

Supplementary Material 5: Table S5 ‘Mock reference’ of 58,582 sequences/contigs based on 202 genotypes of subspecies officinalis built by vsearch clustering. Details in manuscript.

Supplementary Material 6: Table S6 Unfiltered VCF data from 249 genotypes. Details in manuscript.

Supplementary Material 7: Table S7 Unfiltered VCF data from 196 genotypes. Details in manuscript.

Supplementary Material 8: Figure S1 ΔK values for STRUCTURE results based on 249 genotypes and 29,307 SNPs.

Supplementary Material 9: Figure S2 Genome Size (2C) in pg per cluster revealed by HAC based on 249 genotypes and 29,307 SNPs (sorted by means per cluster).

Supplementary Material 10: Figure S3 ΔK values for STRUCTURE results based on 196 genotypes and 9,909 SNPs

Supplementary Material 11: Figure S4 Principal component analysis (PCA) based on 196 genotypes and 9,909 SNPs, presenting PC1 and PC2 showing (A) genotype IDs and (B) cultivar names (and all genotypes as dots). PC = Principal component; in brackets explained variance per PC. Colors indicate classification (light-blue bm = breeding material, green cv = cultivars and commercial material, grey nc = non-classified material, orange wd = wild).

## Data Availability

The raw GBS sequencing data generated and analyzed during this study have been deposited in the European Nucleotide Archive (ENA) at EMBL-EBI under accession number PRJEB106876 (https://www.ebi.ac.uk/ena/browser/view/PRJEB106876). All other relevant data, meta-data of genotypes, unfiltered vcf files, SNP matrices and “mock references” are provided within the supplementary information files of this article.

## Glossary

*altissima*: The subspecies *Melissa officinalis altissima* is frequently briefly called *altissima*
Cluster / Major cluster / Minor cluster / Subcluster / Gene pool: In context of HAC and STRUCTURE the term cluster is used for all well supported clusters within the collection lower than the differentiation between subspecies (see subpopulation). In some contexts, the term major cluster is used to describe a cluster, which comprises several well supported minor clusters (e.g. if STRUCTURE supports several number of clusters). The term subcluster was exclusively used to assess within-cluster diversity for constructing the extended core set, i.e. subclusters were not supported by the set cut-offs for population structure analyses. In the context of untapped (and tapped) genetic diversity, we also used the term gene pool stressing the potential of a cluster or group of clusters for applied breeding.
GBS: Genotyping by sequencing
H_E_ / H_O_: Expected and observed heterozygosity
HAC: Hierarchical agglomerative clustering; this term is simplified used to summarize several methods used in the context of HAC.
MAF: Minor allele frequency
*officinalis*: The subspecies *Melissa officinalis officinalis* is frequently briefly called *officinalis*
PCA /PC: Principal component analysis / principal component
SNP: Single nucleotide polymorphism. For simplicity, SNPs were always called in a diploid biallelic mode disregarding ploidy of the genotypes.
STRUCTURE: Frequently simplified used term for the Bayesian Clustering method named STRUCTURE (Pritchard et al. 2000).
Subcluster: See cluster
Subpopulation: The term subpopulation was exclusively used for the genetic differentiation between subspecies
