# Supplementary figures and images for: Population structure, genetic diversity and core set construction of an international collection of 256 Melissa officinalis genotypes
Source: BMC Plant Biol. 2026 May 1;26:794. doi: 10.1186/s12870-026-08853-8 (PMC13135265; doi:10.1186/s12870-026-08853-8)

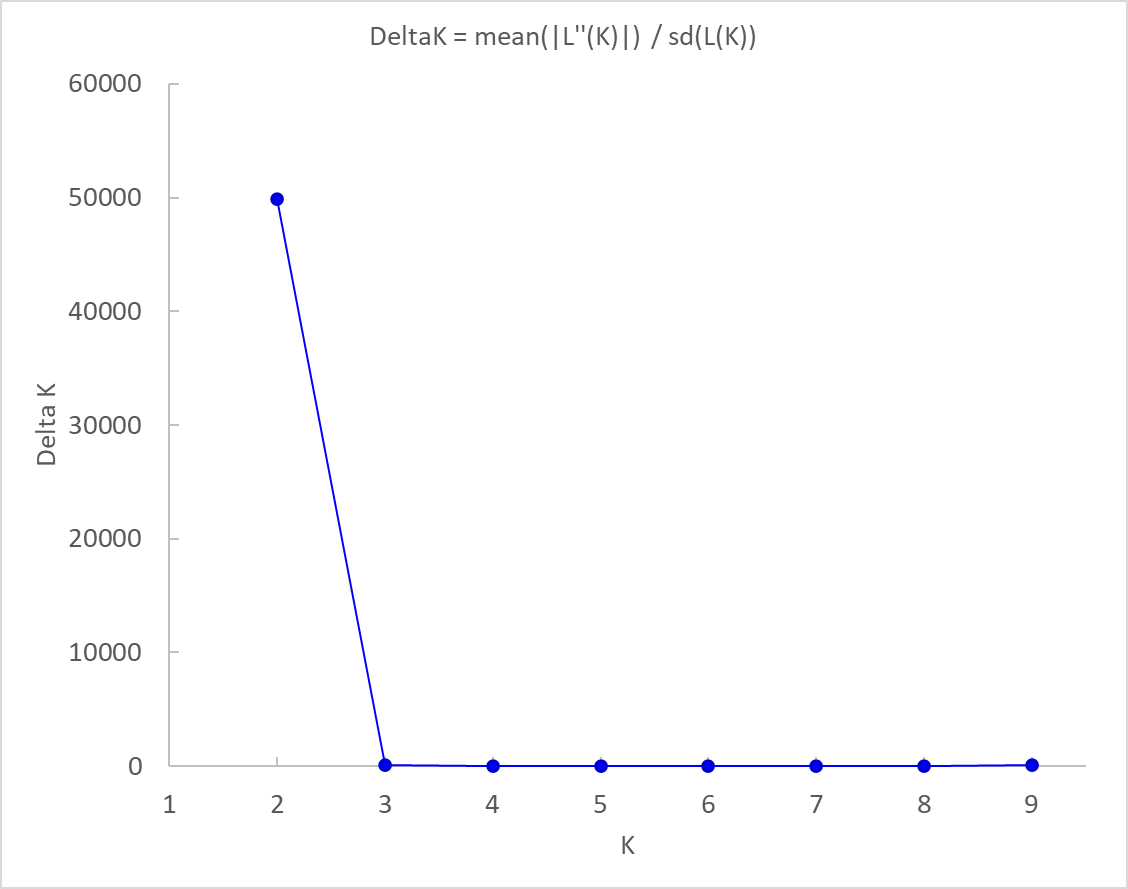

Supplement: Supplementary file 8 — Supplementary Material 8: Figure S1 ΔK values for STRUCTURE results based on 249 genotypes and 29,307 SNPs. [file 12870_2026_8853_MOESM8_ESM.png]

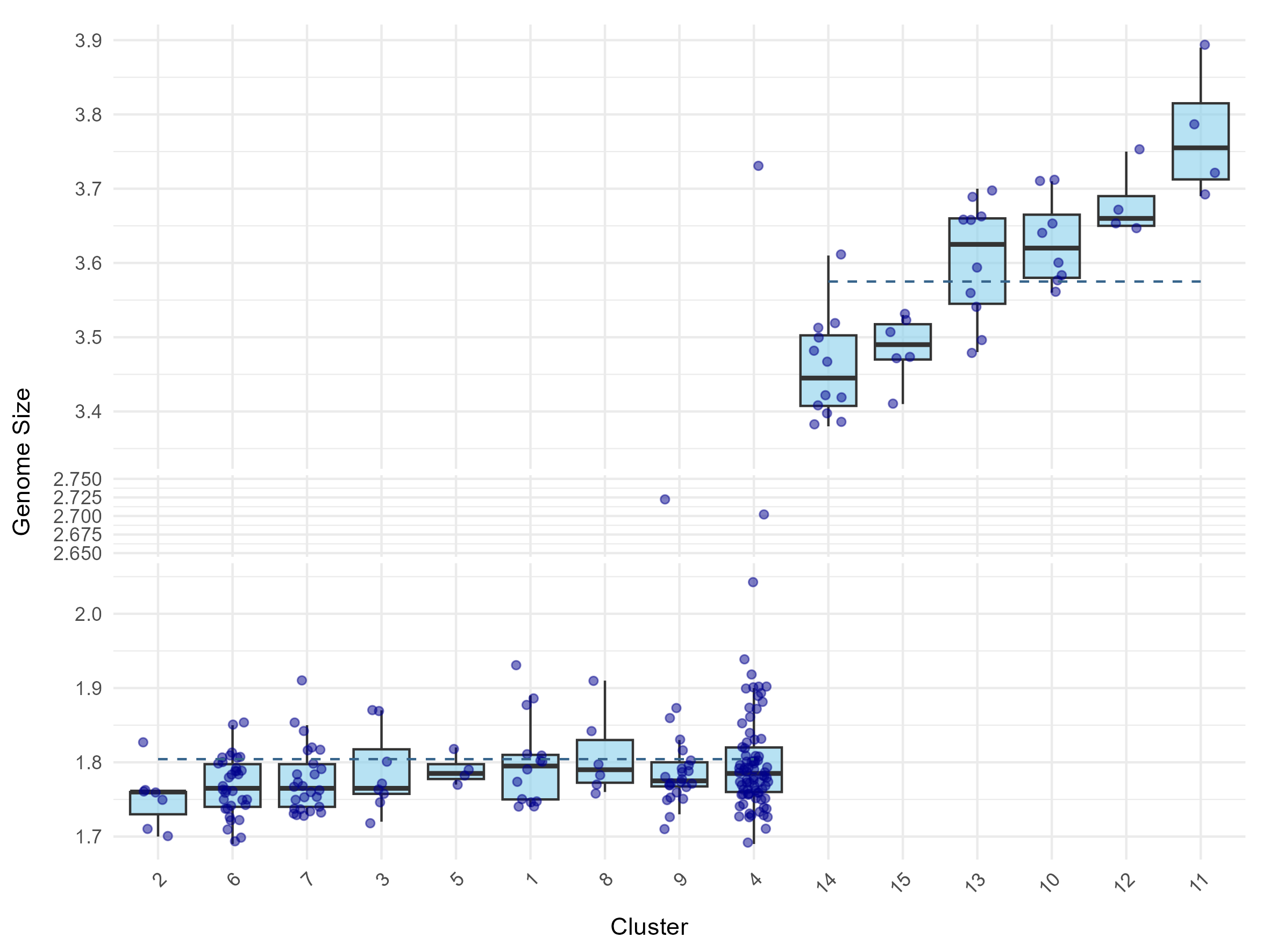

Supplement: Supplementary file 9 — Supplementary Material 9: Figure S2 Genome Size (2C) in pg per cluster revealed by HAC based on 249 genotypes and 29,307 SNPs (sorted by means per cluster). [file 12870_2026_8853_MOESM9_ESM.tif]

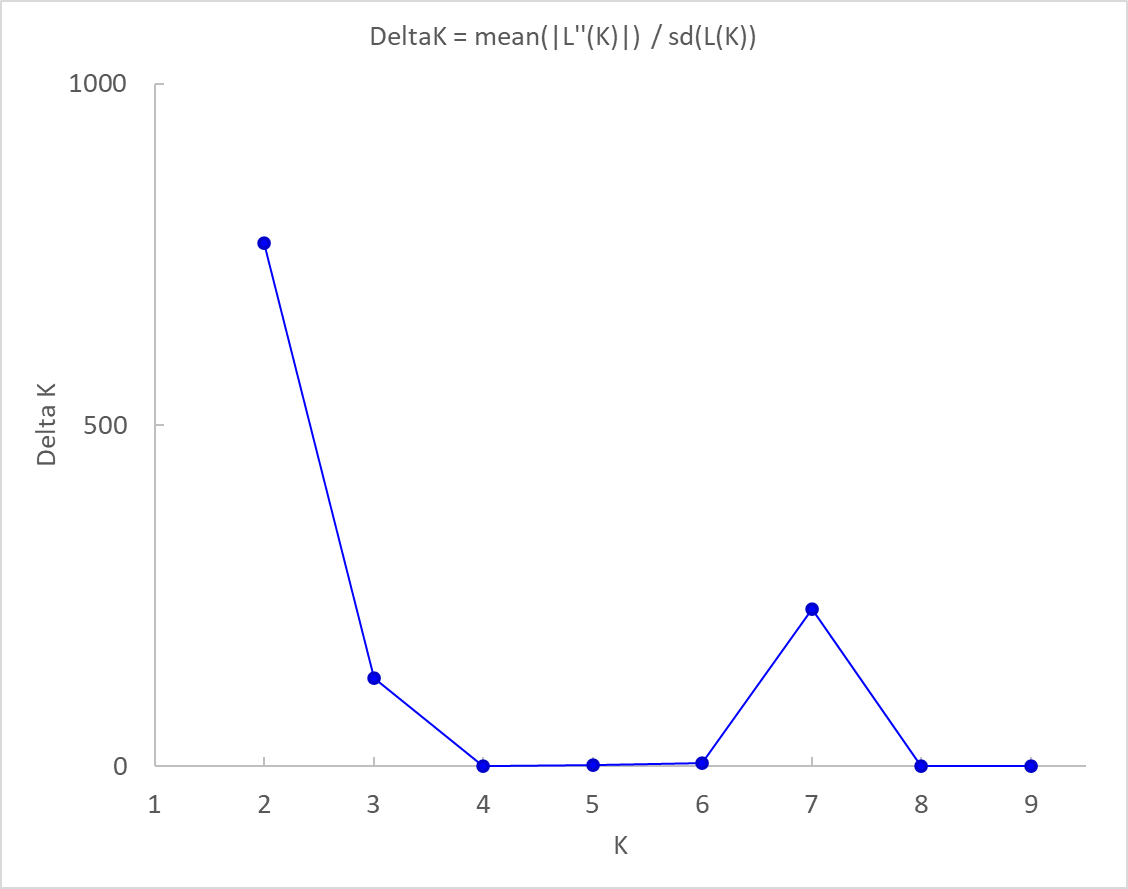

Supplement: Supplementary file 10 — Supplementary Material 10: Figure S3 ΔK values for STRUCTURE results based on 196 genotypes and 9,909 SNPs [file 12870_2026_8853_MOESM10_ESM.png]

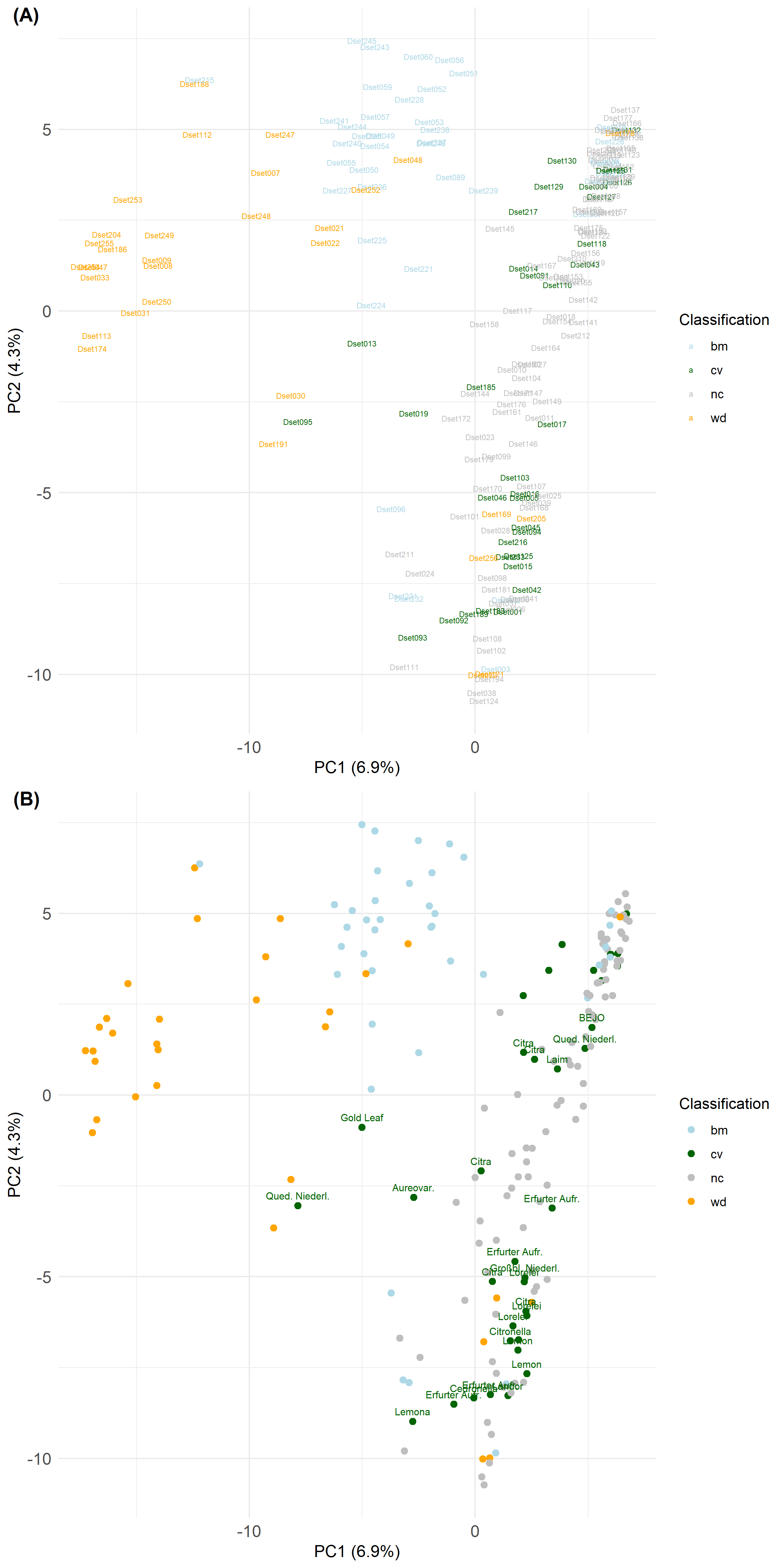

Supplement: Supplementary file 11 — Supplementary Material 11: Figure S4 Principal component analysis (PCA) based on 196 genotypes and 9,909 SNPs, presenting PC1 and PC2 showing (A) genotype IDs and (B) cultivar names (and all genotypes as dots). PC = Principal component; in brackets explained variance per PC. Colors indicate classification (light-blue bm = breeding material, green cv = cultivars and commercial material, grey nc = non-classified material, orange wd = wild). [file 12870_2026_8853_MOESM11_ESM.tiff]
